# Bioinspired Perception and Navigation of Service Robots in Indoor Environments: A Review

**DOI:** 10.3390/biomimetics8040350

**Published:** 2023-08-07

**Authors:** Jianguo Wang, Shiwei Lin, Ang Liu

**Affiliations:** Faculty of Engineering and Information Technology, University of Technology Sydney, Sydney, NSW 2007, Australia; shiwei.lin-1@student.uts.edu.au (S.L.); ang.liu@student.uts.edu.au (A.L.)

**Keywords:** robotic perception, navigation, bioinspired robotics

## Abstract

Biological principles draw attention to service robotics because of similar concepts when robots operate various tasks. Bioinspired perception is significant for robotic perception, which is inspired by animals’ awareness of the environment. This paper reviews the bioinspired perception and navigation of service robots in indoor environments, which are popular applications of civilian robotics. The navigation approaches are classified by perception type, including vision-based, remote sensing, tactile sensor, olfactory, sound-based, inertial, and multimodal navigation. The trend of state-of-art techniques is moving towards multimodal navigation to combine several approaches. The challenges in indoor navigation focus on precise localization and dynamic and complex environments with moving objects and people.

## 1. Introduction

Service robotics has been popular in indoor environments, such as offices, campuses, hotels, and homes. Modern robotic techniques promote robots for autonomous operation in dynamically changing and unstructured environments, and one main technology is to develop autonomous and intelligent robots inspired by biological systems [1].

Biological principles drawn from animals have the potential to produce new ideas for the improvement of robotic techniques. Animals usually have excellent navigation abilities and outstanding robustness, which outperforms current techniques [2]. Animals’ decision-making solutions, action selection, navigation, spatial representation, perception, and explorations make them capable of foraging, homing, and hunting [3].

Robotics aims to achieve complex goals and perform tasks in an unknown environment, so the great inspiration of nature has been promoted. The goal-directed navigation of mobile robots is regarded as animals seeking migration, finding food, and performing similar tasks [4]. With animal perception, cognition, body architecture, and behavior research, biorobotics and neurorobotics bring attention to robotics research. Bioinspired robots can perform several tasks, including transport [5], floor-sweeping [6], security [7], caring [2], and exploration [8].

A robot needs to process sensory information to obtain relevant environmental data by extensive computation or active sensors [9]. Robotic perception is significant for robot navigation to construct an environmental representation with a precise metric map or sensory snapshots [4]. Perception makes a robot aware of its position and how to deviate from an unexpected obstacle blocking its path. Moreover, robots should identify the properties of objects to perform safe and efficient tasks [10].

Reconfigurable robotic systems can respond to the application scenario with their efficiency and versatility, and many robotic platforms are based on a biomimetic design from naturally evolving mechanisms [11]. Intelligent robots can maximize extrinsic and intrinsic rewards and adapt to environmental changes [12]. Robots are created with a high degree of autonomy with autonomous training and adaptation, and exploration and navigation are essential factors.

Several sensors can be used in autonomous vehicles for location and sensing, while indoor environments and buildings are GNSS-denied environments, and virtual-based approaches play a crucial role in high-precision sensing [13]. Tactile [14] and olfactory navigation [15] are also involved in indoor environments. Multimodal navigation is the trend of bioinspired perception and navigation which incorporates the strength of each approach to enhance performance.

This review paper aimed to examine robotic perception and navigation techniques in indoor environments, addressing potential research challenges. The contents are classified by the sensors involved in the approaches. This paper introduces vision-based navigation, remote sensing, multimodel navigation, etc., in Section 2, Section 3, Section 4, Section 5, Section 6, Section 7, Section 8 and Section 9, then provides a discussion and is concluded in Section 10.

## 2. Vision-Based Navigation

### 2.1. Optic Flow

Bioinspired vision has the characteristics of an efficient neural processing, a low image resolution, and a wide field of view [9]. Some vision-based navigation validates biological hypotheses and promotes efficient navigation models by mimicking the brain’s navigation system [16]. The main research directions of the visual-based approaches are optic flow and SLAM. They are used to explore or navigate unknown areas. Vision-based navigation is popular in an indoor environment to detect the surroundings and obstacles. Sensor fusion and deep learning improve the performance and provide more reliable decisions.

**Figure 1:**
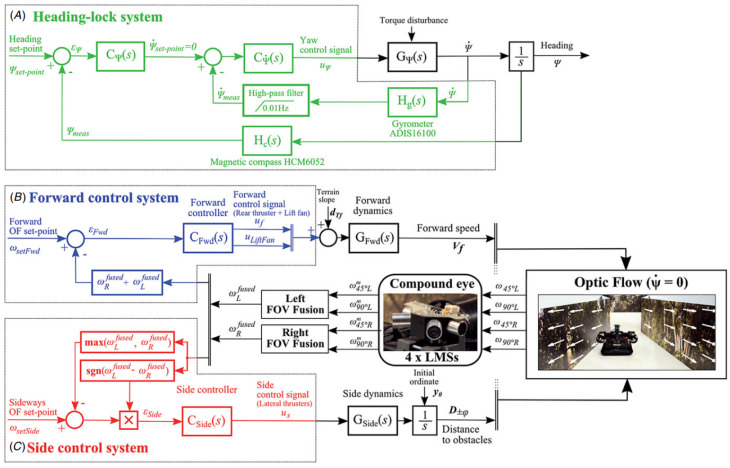
Feedback loops consisting of a heading-lock system and an optic-flow-based autopilot, which uses a forward and a side control loop with a dual lateral optic flow regulator [17].

Roubieu et al. [17] presented a biomimetic miniature hovercraft to travel along various corridors with the optic flow, which used a minimalistic visual system to measure the lateral optic flow for controlling robots’ clearance from the walls and forward speed in challenging environments, as shown in Figure 1. The restricted field of view is the limitation of the visual perception systems, which may not perform successful navigation in complex environments, such as challenging corridors.

**Figure 2:**
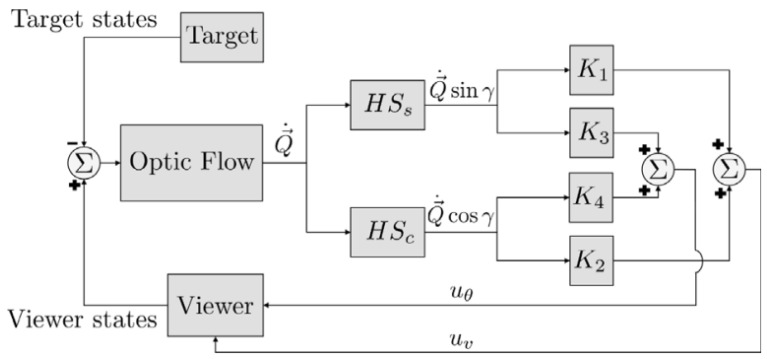
Visual feedback model of an optic-flow enrichment to provide the difference between the viewer and target insect [19].

A collision avoidance model based on correlation-type elementary motion detectors with the optic flow in simple or cluttered environments was proposed in [18]. It used the depth information from optic flow as input to the collision avoidance algorithm under closed-loop conditions, but the optimal path could not be guaranteed. Yadipour et al. [19] developed an optic-flow enrichment model with visual feedback paths and neural control circuits, and the feedback car provided the relative position regulation. The visual feedback model was a bioinspired closed loop, as shown in Figure 2. Dynamics-specialized optic-flow coefficients would be required as an improvement.

**Figure 3:**
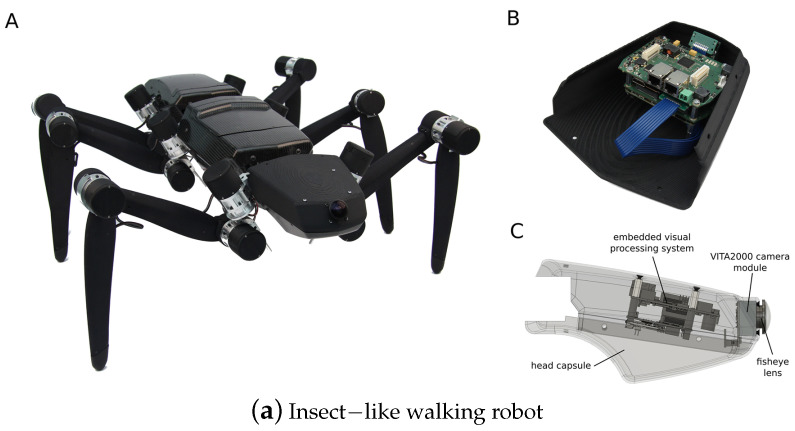

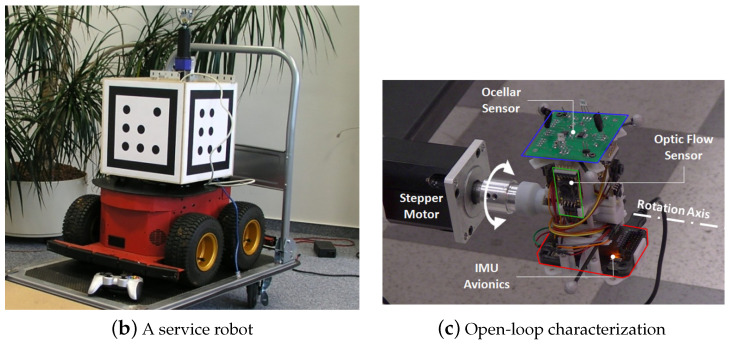
(**a**) Insect-like walking robot with a bottom view and rendered side view of the front segment. (A) is the hexapod walking robot. (B) is the rendered side view. (C) is the front segment of the robot. It was inspired by the stick insect and adopted the orientation of its legs’ joint axes and the relative positions of its legs [9]. (**b**) A service robot with an omnidirectional vision system and a cube for flexible and robust acquisition [2]. (**c**) Open-loop characterization [32].

A control-theoretic framework was developed for directional motion preferences, and it processed the optic flow in lobula plate tangential cells [20]. It simplified the operation of the control architectures and formalized gain synthesis tasks as linear feedback control problems and tactical state estimation. However, it assumed an infinite tunnel environment and small perturbations. Resource-efficient visual processing was proposed in [9] with insect-like walking robots such as the mobile platforms shown in Figure 3a, which consisted of image preprocessing, optic flow estimation, navigation, and behavioral control. It supported controlling the collision avoidance behavior by leveraging optimized parallel processing, serialized computing, and direction communication.

However, the major challenges of these perception approaches are dealing with dynamic obstacles. Although the feedback control provides robust operation, dynamic obstacles are not considered or successfully handled. Dynamic environments involve moving obstacles that significantly decrease performance or cause ineffective operation. Detection and tracking of dynamic obstacles still remain difficult for bioinspired perception and navigation. For optic flow, the configurations of the coefficient for a dynamic environment or combination of other sensors are presented as considerations.

An event-based neuromorphic system senses, computes, and learns via asynchronous event-based communication, and the communication module is based on the address-event representation (AER) [21]. Action potentials, known as “spikes,” are treated as digital events traveling along axons. Neuromorphic systems integrate complex networks of axons, synapses, and neurons. When a threshold is exceeded, a neuron sends the event to other neurons and fires an action potential [22]. The advantages of an event-based visual system include a low power and latency and a high dynamic range and temporal resolution. A spiking neural network (SNN) is suitable for processing the sparse data generated by event-based sensors from spike-based and asynchronous neural models [23].

A gradient-based optical flow strategy was applied for neuromorphic motion estimation with GPU-based acceleration, which was suitable for surveillance, security, and tracking in noisy or complex environments [7]. The GPU parallel computation could exploit the complex memory hierarchy and distribute the tasks reasonably. Moreover, a single-chip and integrated solution was presented with wide-field integration methods, which were incorporated with the on-chip programmable optic flow, elementary motion detectors, and mismatch compensation [24]. It achieved real-time feedback with parallel computation in the analog domain.

Paredes-Valles et al. [23] proposed a hierarchical SNN with the event-based vision for a high-bandwidth optical flow estimation. The hierarchical SNN performed global motion perception with unsupervised learning from the raw stimuli. The event camera was categorized as a dynamic vision sensor and used the AER as a communication protocol. An adaptive spiking neuron model was used for varying the input statistics with a novel formulation. Then, the authors used a novel spike-timing-dependent plasticity implementation and SNN for the hierarchical feature extraction.

Another optical estimation with an event-based camera, such as a dynamic and active-pixel vision sensor, was proposed by Zhu et al. [25], which presented a self-supervised neural network called EV-FlowNet. A novel, deep learning pipeline fed the image-based representation into a self-supervised neural network. The network recorded data from the camera and was trained without manual labeling. However, the challenging lighting and high-speed motions remained challenging for the neural network.

Two automatic control systems were developed with optical flow and optical scanning sensors, and they could track a target inspired by insects’ visuomotor control systems [26]. The visuomotor control loop used elementary motion detectors to extract information from the optical flow. Li et al. [27] characterized an adaptive peripheral visual system based on the optical-flow spatial information from elementary motion detector responses. The complementary approach processed and adapted the peripheral information from the visual motion pathway.

de Croon et al. [28] developed a motion model combined with the optic flow to accommodate unobservability for attitude control. Optic flow divergence allowed independent flight and could improve redundancy, but it would need more sensors to improve observability. For measuring optical flow, comparative tests of optical flow calculations were presented in [29] with the contrast of “time of travel”. Two time-of-travel algorithms relied on cross-correlation or thresholding of adjacent bandpass-filtered visual signals.

**Figure 4:**
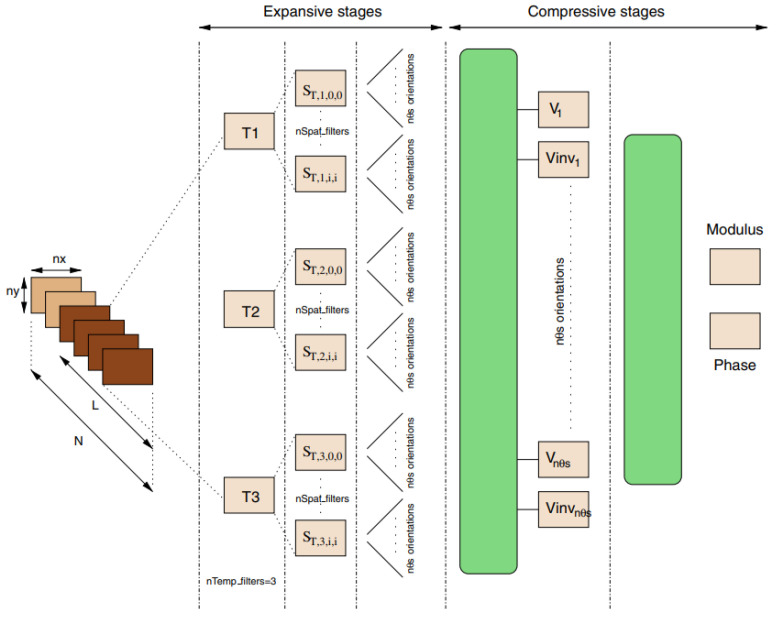
The multichannel gradient model with temporal and spatial filtering, steering, speed and velocity, and direction [31].

Feedback loops were designed to employ the translational optic flow for collision-free navigation in an unpredictable environment [30]. The optic flow could be generated as the related motion between the scene and the observer, and the translational optic flow was for short-range navigation. It used the relative linear speed and the distance as the ratio. Igual et al. [31] promoted a robust gradient-based optical flow algorithm for robust motion estimation. It could be implemented for tracking or biomedical assistance in a low-power platform, and real-time requirements were satisfied by a multicore digital signal processor, as shown in Figure 4. However, it lacked detailed measurements about power consumption and real measurements for the system and core levels.

Zufferey et al. [33] designed an ultralight autonomous microflier for small buildings or house environments based on the optic flow with two camera modules, rate gyroscopes, a microcontroller, a Bluetooth radio module, and an anemometer. It could support lateral collision avoidance and airspeed regulation, but the visual textures could be further enhanced. Another vision-based autopilot was later presented with obstacle avoidance and joint speed control via the optic flow in confined indoor environments [34], which traveled along corridors by controlling the clearance and speed from walls. The visuomotor control system was a dual-optic-flow regulator.

**Figure 5:**
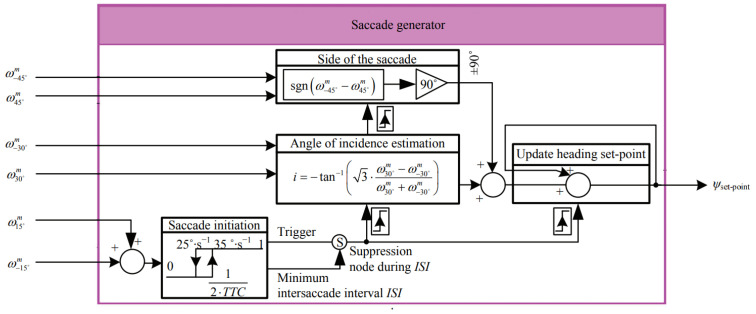
Description ofthe saccade system [35].

Ref. [35] introduced a bioinspired autopilot that combined intersaccadic and body-saccadic systems, and the saccadic system avoided frontal collisions and triggered yawing body saccades based on local optic flow measurements. The dual OF regulator controlled the speed via an intersaccadic system that responded to frontal obstacles, as shown in Figure 5. Ref. [36] provided guidelines of navigation based on a wide-field integration of the optic flow, and the wide-field integration enabled motion estimation. The system was lightweight and small for micro air vehicles with low computation requirements. A gyro sensor was combined with the wide-field integration for the estimation.

Serres et al. [37] introduced an optic-flow-based autopilot to avoid the corridors and travel safely with a visuomotor feedback loop named Lateral Optic Flow Regulation Autopilot, Mark 1. The feedback loop included a lateral optic flow regulator to adjust the robot’s yaw velocity, and the robot was endowed with natural pitch and roll stabilization characteristics to be guided in confined indoor environments. Ref. [38] developed an efficient optical flow algorithm for micro aerial vehicles in indoor environments and used the stereo-based distance to retrieve the velocity.

From the mentioned navigation approaches, the challenges also include hardware and logic limitations and the implemented sensor algorithms. For example, the motion detection architecture [27] and optimal spectral extension [7] should be improved. Obstacle avoidance logic and control parameters should be investigated more in complex environments [33,35,38]. The challenging lighting environments should also be considered [25]. The optimal implementation of algorithms is also a limitation, which may not be satisfied by dynamics models [19]. Sensor fusion would be helpful to improve observability [17,28].

### 2.2. SLAM

**Figure 6:**
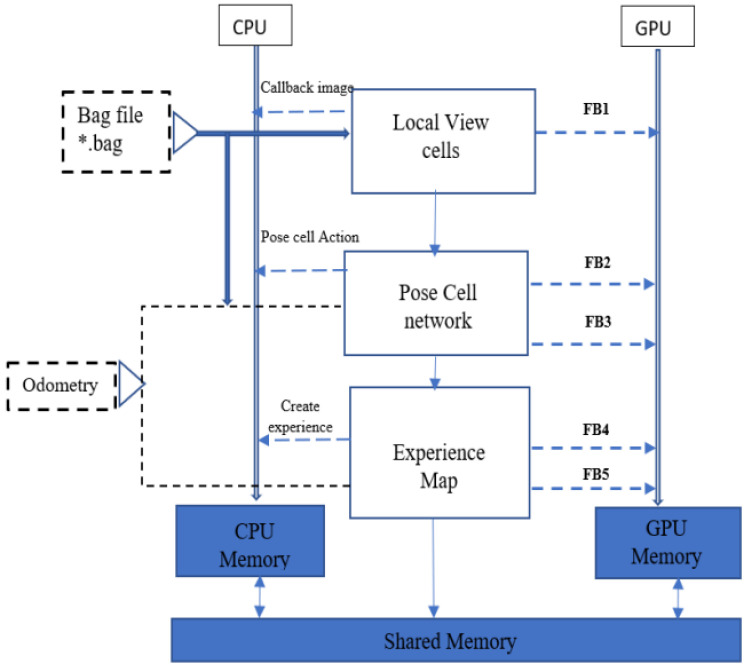
Schematic representation of functional blocks in the bioinspired SLAM, and ∗.bag represented bag file [39].

A simultaneous localization and mapping system (SLAM) can construct a map and calculate the pose simultaneously, so it is implemented with different sensors for localization [39]. A heterogeneous architecture was introduced for a bioinspired SLAM for embedded applications to achieve workload partitioning in [39], as demonstrated in Figure 6. It used local view cells and pose cell networks for the image processing to improve time performance, although it could not achieve processing on the fly.

Vidal et al. [40] presented a state estimation pipeline for visual-based navigation to combine standard frames, coupled manner events, and inertial measurements for SLAM. The hybrid pipeline provided an accurate and robust state estimation and included standard frames for real-time application in challenging situations. Refocused events fusion was proposed with multiple event cameras for outlier rejection and depth estimation to fuse disparity space images [41], and it performed a stereo 3D reconstruction of SLAM and visual odometry. The limitation of that research was the camera tracking algorithm; if the proposed method was integrated with a tracking algorithm, a full event-based stereo SLAM could be achieved.

Another framework based on an event camera was proposed in [42] with CNNs. Its solution only relied on event-based camera data and used the neural network for the relative camera depth and pose. The event data were more sensitive to the pose when involving rotations. However, the SLAM solution was limited to an offline implementation, and the used dataset was under a static environment. The proposed networks also had the challenges of parameter size.

Pathmakumar et al. [43] described a dirty sample-gathering strategy for cleaning robots with swarm algorithms and a geometrical feature extraction. The approach covered identified dirt locations for cleaning and used geometric signatures to identify dirt-accumulated locations. It used SLAM to get the 2D occupancy grid and ant colony optimization (ACO) for the best cleaning auditing path. Machine-learning-based or olfactory sensing techniques were the next step. An efficient decentralized approach, an immunized token-based approach [44], was proposed for an autonomous deployment in burnt or unknown environments to estimate the severity of damage or support rescue efforts. It used SLAM to detect the environment, and the robots carried wireless devices to create communication and sensing coverage.

Jacobson et al. [45] introduced a movement-based autonomous calibration technique inspired by rats based on Open RatSLAM, which performed self-motion and place recognition for multisensory configurations. It used a laser, an RGB and range sensor, cameras and sonar sensors for online sensor fusion, and weighting based on the types of environments, including an office and a campus. RatSLAM was improved to enhance its environmental adaptability based on the hue, saturation, and intensity (HSI) color space that handles image saturation and brightness from a biological visual model [46]. The algorithm converted the raw RGB data to the HSI color space via a geometry derivation method, then used a homomorphic filter and guided filtering to improve the significance of details.

**Figure 7:**
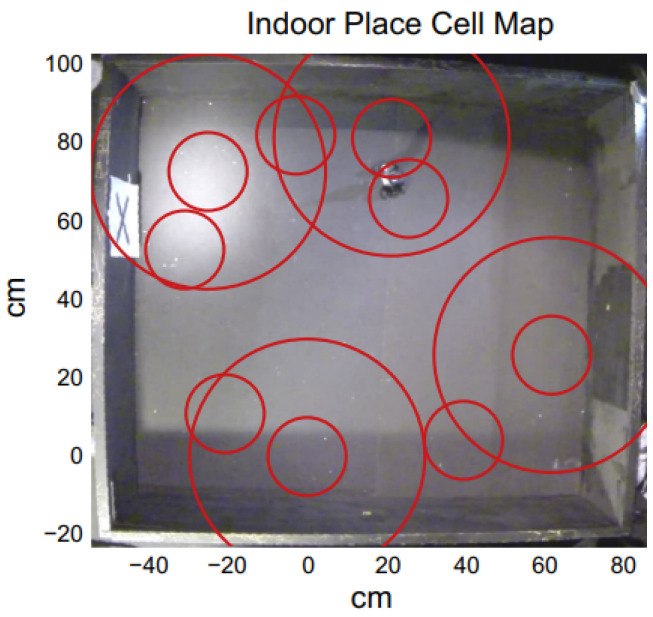
Indoor place cell map, and the red circles are place cell firing fields [47].

A hierarchical look-ahead trajectory model (HiLAM) was developed by combining RatSLAM and HiLAM, which incorporated media entorhinal grid cells and a hippocampus and prefrontal cortex. It employed RatSLAM for real-time processing and developed the hybrid model based on a serialized batch-processing method [47]. Figure 7 shows an indoor place cell map. A slow feature analysis network was applied to perform visual self-localization with a single unsupervised learning rule in [2], and it used an omnidirectional mirror and simulated rotational movement to manipulate the image statistics as shown in Figure 3b. It enhanced the self-localization accuracies with LSD-SLAM and ORB-SLAM, but it could have difficulties handling the appearance changes.

The limitations of the proposed SLAM approaches include a lack of support of real-time image processing [39] or image detection [46] and difficulties in handling environmental changes [2]. These make such systems respond slowly or not respond to environmental changes. Moreover, they can cause failures in navigation or make incorrect decisions if visual disturbances exist. More powerful strategies, such as machine learning or neural networks, could be combined to overcome these issues, but they require a comprehensive dataset generation [43]. Parallel processing could also be used as a solution [39].

### 2.3. Landmark

Sadeghi Amjadi et al. [48] put forward a self-adaptive landmark-based navigation inspired by bees and ants, and robots located the cue by their relative position. The landmark of this navigation method used a QR code to identify the environment and employed a camera for the relative distance by perspective-n-point algorithms. It was adaptive to environmental changes but lacked any consideration of the presence of stationary and moving objects. Ref. [49] compared landmark-based approaches, including average landmark vector, average correctional vector, and distance estimated landmark vector approaches, and proposed a landmark vector algorithm using retinal images. The results showed that the distance estimated landmark vector algorithm performed more robust homing navigation with occluded or missing landmarks than others.

Maravall et al. [5] designed an autonomous navigation and self-semantic location algorithm for indoor drones based on visual topological maps and entropy-based vision. It supported robot homing and searching and had online capabilities because of metric maps and a conventional bug algorithm. The implementation of other situations should be analyzed further. Ref. [50] introduced a pan–tilt-based visual system as a low-altitude reconnaissance strategy based on a perception-motion dynamics loop and a scheme of active perception. The dynamics loop based on chaotic evolution could converge the periodic orbits and implement continuous navigation. The computational performance was not analyzed. A new data structure for landmarks was developed as a landmark-tree map with an omnidirectional camera, and it presented a novel mapping strategy with a hierarchic and nonmetric nature to overcome memory limitations [51]. However, its feature tracker could not support long distances.

Although some self-adaptive frameworks were proposed, dynamic obstacles were not considered [48,49,51]. Object tracking is also a bottleneck for the landmark-based approach if the landmark is moving over large distances. Some papers’ implementations or experiments are difficult to conduct due to the situations under certain environments or hardware limitations [5,48]. The computational performance of the system should develop a measurement strategy.

### 2.4. Others

Proper vergence control reduces the search space and simplifies the related algorithms, and a bioinspired vergence control for a binocular active vision system was introduced in [8]. It controlled the binocular coordination of camera movements to offer real-world operation and allow an exploration of the environment. Salih et al. [52] developed a vision-based approach for security robots with wireless cameras, and the approach used a principal component analysis algorithm for image processing, a particle filter for images, and a contour model. The system could recognize objects independently in all light conditions for frame tracking.

**Figure 8:**
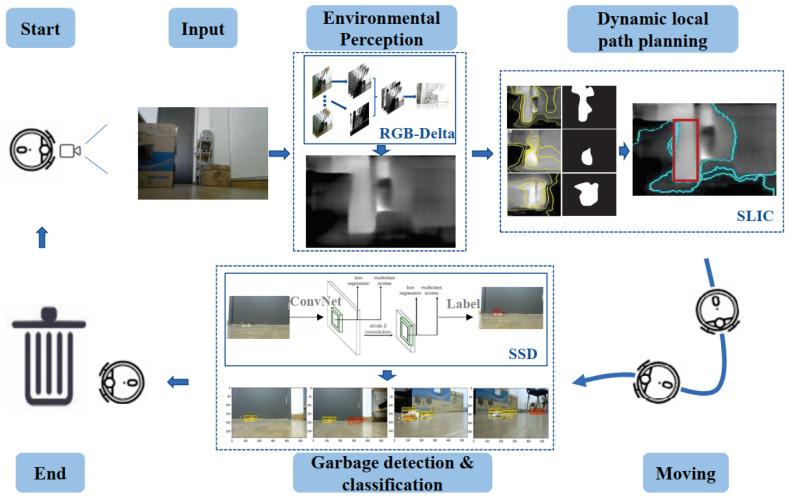
Visual-based navigation for floor-sweeping [6].

A camera-based autonomous navigation system was conceptualized for floor-sweeping robots in [6], including inspection, targeting, environment perception, local path planning, and directional calibration, as demonstrated in Figure 8. It achieved image processing and map planning by a superpixel image segment algorithm, but it could interfere with light. Cheng et al. [53] designed a distributed approach with networked wireless vision sensors and mosaic eyes. It performed localization, image processing, and robot navigation with multiple modules and obtained real-time obstacle coordinates and robot locations. The limitation of the work was the coordination of multiple cameras; the framework could be further improved for mapping the images to a workspace.

Li et al. [54] developed a parallel-mechanism-based docking system with the onboard visual perception of active infrared signals or passive markers. The modules performed docking based on relative positioning, and the self-assembly robot could react to different environments, such as stairs, gaps, or obstacles. However, the applications of the docking system were limited without a positioning system. Ref. [55] conceptualized a lightweight signal processing and control architecture with visual tools and used a custom OpenGL application for real-time processing. The novel visual tool was inspired by a vector field design for exploiting the dynamics and aiding behavioral primitives with signal processing. The control law and schemes could be improved in that framework.

Boudra et al. [56] introduced a mobile robot’s cooperation and navigation based on visual servoing, which controlled the angular and linear velocities of the multiple robots. The interaction matrix was developed to combine the images with velocities and estimate the depth of the target and each robot, although it could not be applied to 3D parameters. Ahmad et al. [57] developed a probabilistic depth perception with an artificial potential field (APF) and a particle filter (PF), formulating the repulsive action as a partially observable Markov decision process. It supported 3D solutions in real time with raw sensor data and directly used depth images to track scene objects with the PF. The model could not address the problem of dynamic obstacles or dynamic prediction.

An ocular visual system was designed for a visual simulation environment based on electrophysiological, behavioral, and anatomical data with a fully analog-printed circuit board sensor [32]. The model used a Monte Carlo simulation for linear measurements, an open-loop sensor characterization, and close-loop stabilizing feedback, as displayed in Figure 3c. Nguyen et al. [58] described an appearance-based visual-teach-and-repeat model to follow a desired route in a GPS-denied environment. The repeated phases made the robot navigate along the route with reference images and determine the current segment by self-localization by sped-up robust features to match images. An effective fusion of sensors could be further required.

A probabilistic framework was presented with a server–client mechanism using monocular vision for terrain perception by a reconfigurable biomimetic robot [59]. GPGPU coding performed real-time image processing, and it supported the unsupervised terrain classification. The perception module could be extended with an IMU sensor. Montiel-Ross et al. [60] proposed a stereoscopic vision approach without depth estimation, which used an adaptive candidate matching window for block matching to improve accuracy and efficiency. The global planning was achieved through simple ACO with distance optimization and memory capability, and the obstacle and surface ground detection were achieved by hue and luminance.

Aznar et al. [61] modeled a multi-UAV swam deployment with a fully decentralized visual system to cover an unknown area. It had a low computing load and provided more adaptable behaviors in complex indoor environments. An ad hoc communication network established communications within the zone. A V-shaped formation control approach with binoculars was applied to the robotic swarms for unknown region exploration in [62] with a leader–follower structure. The formation control applied a finite-state machine with a behavior-based formation-forming approach, considering obstacle avoidance and anticollision. However, the physical application was a challenge due to the devices, such as the physical emitter or sensor [32,59,62]. The indirect communication between a swarm of robots or a sensor-based communication protocol is hard to achieve [61,62].

## 3. Remote Sensing Navigation

Besides visual sensors, remote sensing techniques also play a crucial role in robot perception in the indoor environment. The primary research of remote sensing approaches is focused on laser, infrared sensors, and radar. Some papers implement metaheuristic algorithms or machine-learning-based methods for dynamic systems. Sensor fusion is also an effective way for remote sensing techniques to improve accuracy.

Efficient sensorimotor convergence approaches are developed by animals, which allow a fast processing of huge, spatially distributed measurements into signals. Ref. [63] proposed a bioinspired wide-field integration framework based on sensorimotor convergence with a LiDAR sensor. Its advantage was its computational simplicity and robustness against additive Gaussian sensor noise or occlusions in the measurements. However, it had limitations when working with unfiltered points and unknown spatial distributions. The data quality could not be guaranteed and it could not work with unstructured data.

A bioinspired reconfigurable robot was developed for navigation and exploration to work with laser-induced sensors [64]. Its control system was hierarchical and consisted of body-level and low-level controllers to generate control directives and paths for each component, then it generated reference points. It achieved awareness and sensing by production sensors to merge data in real time [64]. Jiang et al. [65] proposed a local path planning algorithm based on 2D LiDAR for reactive navigation in corridor-like environments. The LiDAR data were converted to a binary image first to extract the skeleton via a thinning algorithm; then, the center line was extracted to smooth the obtained roadway. However, the navigation approach was only conducted in a limited simulation. It could not realize reactive navigation, so integrated sensors would be needed.

Romeh and Mirjalili [66] described a combined metaheuristic salp swarm algorithm for multirobot exploration to reduce uncertainties and search for a space with a laser range scanner. The coordinated multirobot exploration determined adjacent cells with a utility and cost, then optimized the path using the salp swarm algorithm. The limitation was that the robot would visit an explored area more than once; a multiobjective algorithm should overcome this problem when searching for a new space. Ref. [67] provided a robot localization technique using a nonlinear evolutive filter, named evolutive localization filter, via the Markov chain behavior, with a robot equipped with a laser range finder. It could deal with non-Gaussian noise, sensor characteristics, and arbitrary nonlinear system dynamics.

Le et al. [68] developed a modified A-star zigzag global planner with an integrated laser sensor for a cleaning robot. The algorithm covered the narrow spaces by waypoints, aiming to maximize the coverage area. The adaptative Monte Carlo localization used particle filters to filter out the odometry’s noise and get the real-time position from the visual sensor data. A simple chemical signal model was proposed to find the recharging stations for robots, reducing the exploration times [69]. It adopted the ant foraging swarm algorithm and a perturbed Markov chain for processing dynamics with infrared sensors, but the applied situation was only limited to one charging point.

Saez-Pons et al. [70] introduced a social potential field framework with a LiDAR range finder for search and rescue or fire services in a warehouse, which supported human–robot or multirobot teams with potential functions. The control model could exercise collision avoidance, formation generation and keeping, and obstacle avoidance behaviors. Martinez et al. [71] explored an unknown polygonal and connected environment by a motion policy based on a complete exploration strategy and simple sensor feedback with the shape of a disc. It directly mapped to the control from the observation with omnidirectional sensors such as two laser range finders. The robot dynamics was dealt with by a practical hybrid control scheme to maintain the linear and angular velocities. However, these approaches were applied under specific conditions. The robots’ visibility domains and arbitrary sizes were limited. The formation of the environment and the execution based on feedback were restricted.

Additionally, Arvin et al. [72] presented a low-cost and adaptable robotic platform for teaching and education with infrared proximity sensors. The hardware functionality included communication, actuation, power management, and characterized sensory systems, and the motion control used the encoders’ data as the input to the closed-loop motion control. The next stage focused on pheromone communication and fault-tolerant control.

A generic fault-detection system was presented with infrared proximity sensors, range-and-bearing sensors, and actuators to detect faults with a low false positive rate, and it achieved long-term autonomy for multirobot systems [73]. The homogeneous and heterogeneous swarm behaviors were considered for the robot swarm, which detected injected faults, although the real experiment was not conducted, and the fault-detection system was limited to a small swarm of robots.

A swarm behavior algorithm was described based on influence, attraction, and repulsion with a pack of robots and sensory limitations in [74]. The robots used ultrasonic, infrared sensors, and light-dependent resistance. Gia Luan and Truong Thinh [75] implemented a wave-based communication mechanism inspired by slime mold aggregation to measure the cluster size for a swarm of robots with infrared sensors. The robot could grasp the cluster size and detect a desired cluster, then approach the cluster by the average origin of wave method. It was hard to adapt these approaches’ control parameters to the swarm behavior, which may result in some blind spots or dead zones. The robustness of the models could not be assessed, and the experiments were only for the simple environment under simulation.

A cognitive, dynamic sensing system based on radar and sonar perception for target recognition and classification was designed in [76]. It exploited a memory-driven perception to interact and navigate with man-made echoic sensors for control problems. However, the sensing system’s performance was low and could not categorize information in challenging scenarios. Ordaz-Rivas et al. [77] proposed a form of steering robot based on local behavior rules, including attraction orientation, repulsion, and influence. The influence emphasized the principal task, the behavior rules determined the formation of the swarm, and a specific signal or perception was associated with a specific task. It used proximity sensors, light-dependent resistors, and ultrasonic sensors for detecting obstacles. Influence parameters and repulsion–attraction–orientation could be future considerations.

Bouraine and Azouaoui [78] demonstrated a tree expansion algorithm based on particle swarm optimization (PSO) dubbed PASSPMP-PSO, which supported objects moving at high speed with an arbitrary path and dealt with sensors’ field-of-view limitations. It was based on the execution of regular updates of the environment and a periodic process that interleaved planning. Its safety issues could be considered further. Ref. [79] designed a robot’s control mechanism based on PSO and a proportional–integral–derivative controller with distance sensors for service robot navigation in complex environments. The ESP32 microcontroller performed the motion planning and had execution capabilities. The perception capabilities were limited, and the motion planning algorithm could be improved. Additional sensors may be required for higher adaptability and performance.

## 4. Tactile-Sensor-Based Navigation

Insects or animals sense the environment by touching their surroundings, and they have skin covering their arms, legs, bodies, or antennas. A tactile sensor is usually used for terrain identification and navigation. It is a typical approach to a design bioinspired by skins or whiskers. It can determine the properties of terrain and avoid obstacles. A multimodal approach is usually implemented for tactile sensors.

**Figure 9:**
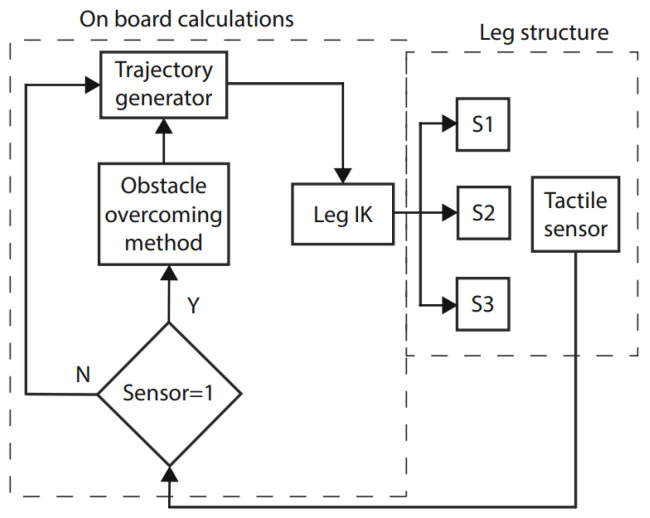
Diagram of a tactile-based obstacle overcoming method [14].

A hybrid tactile-sensor-based method was proposed with a generative recurrent neural network for obstacle overcoming, and it aimed to provide solutions for multilegged service robots [14]. The robot could move in an unstructured and uncertain environment by touching the obstacle and calculating the new leg path parameters, as shown in Figure 9. A new architecture based on spiking neurons was proposed for motor-skill learning for insectlike walking robots [80]. It involved the mushroom bodies of the insect brain, modeled as a nonlinear recurrent SNN. It could memorize the time evolutions of the controller to improve the existing motor primitives.

**Figure 10:**
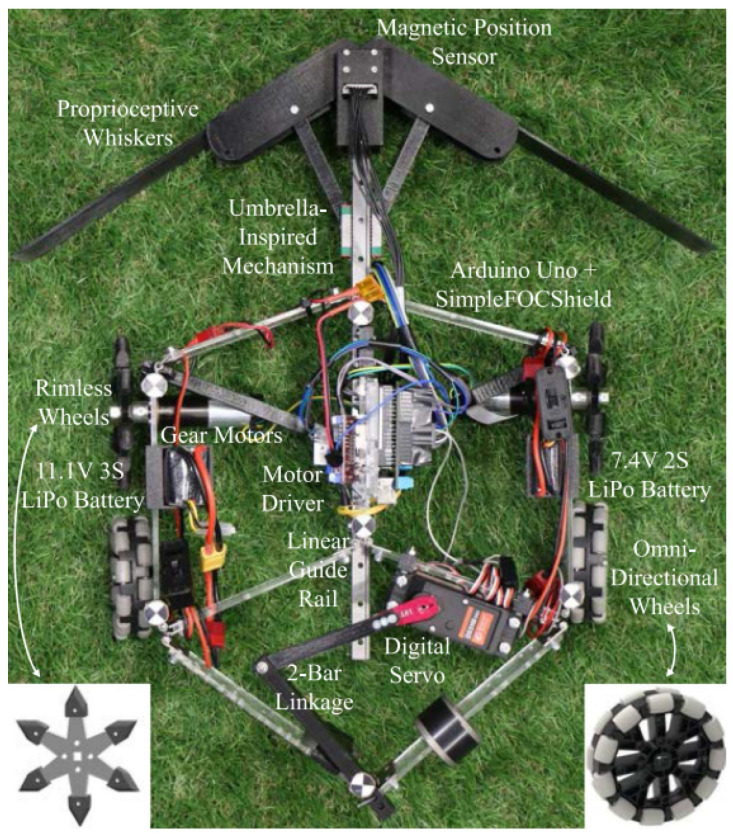
Design of a deformable robot with sensors [82].

Xu et al. [81] designed a triboelectric palmlike tactile sensor with a granular texture in the palms, and it maintained stable performance in real time via the triboelectric nanogenerator technology and palm structure. Its inner neural architecture offered clues about tactile information and was used for tactile perception. Ref. [82] proposed a whisker-based feedback control for a bioinspired deformable robot to perform shape adjustment to traverse spaces smaller than its body, as in Figure 10. Its shape adjustment could provide proprioceptive feedback for real-time estimation to balance stability, locomotion, and mobility.

**Figure 11:**
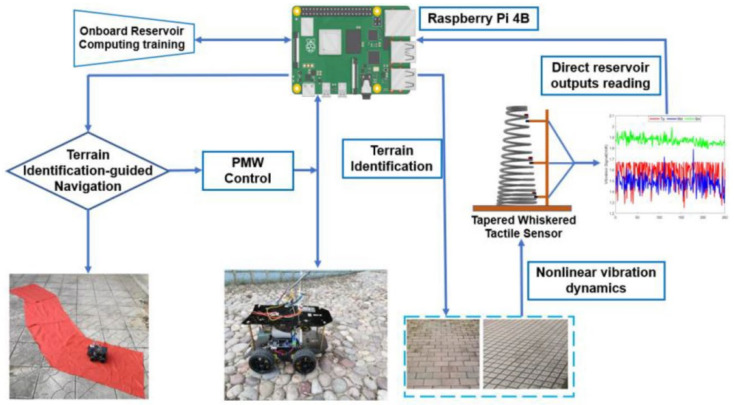
Workflow of whisker-based reservoir computing systems [83].

Another whisker-based system was designed for terrain-recognition-based navigation with real-time performance, which used a tapered whisker-based reservoir computing system [83]. The system provided a nonlinear dynamic response and directly identified frequency signals; the workflow is demonstrated in Figure 11. The limitation of the whisker-based approach was the classification accuracy of the terrain; the whisker sensors could be further improved.

## 5. Olfactory Navigation

Animals employ the sense of smell for mating, inspection, hunting, and recognition, even though smell is not the principal perception mechanism. Odor molecules through olfaction can be used for strategies with a robot to find the direction of odor trails to locate the origin of a fire or a toxic gas leak [15]. The main research directions of olfactory navigation include odor source localization and tracking, odor recognition, and search. The olfactory sensors are usually combined with other sensors or AI techniques for odor mapping and navigation.

An odor tracking algorithm was employed with genetic programming for tracking the odor plume, and the genetic programming was used as a learning technique platform for the odor source localization algorithm. However, it lacked obstacle-avoidance techniques [15]. A search-based algorithm to detect a gas source localization based on the silkworm moth integrated a repulsion function and worked in surface obstacle regions [84]. It used gas sensors for odor stimuli and a distance sensor for object detection. The approach was limited to searching in a two-dimensional environment; an active searching algorithm should be integrated.

**Figure 12:**
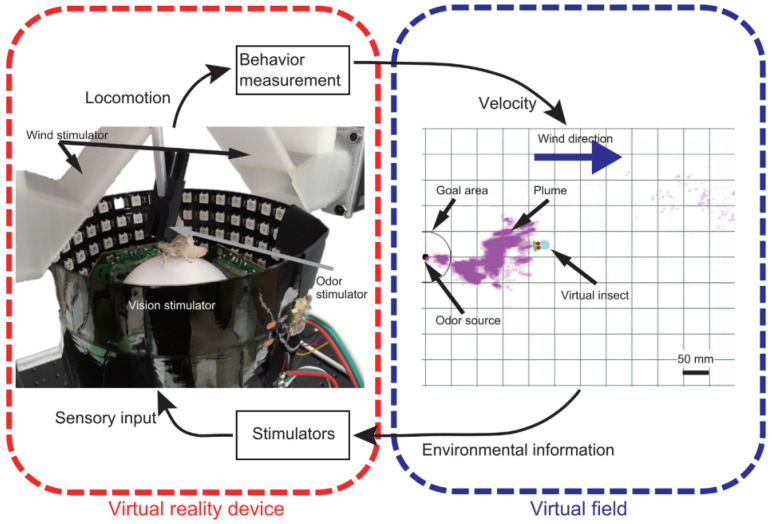
Odor-source search fields with the initial heading, the result of paths, and heading angle histograms [86].

Ojeda et al. [85] introduced a framework for integrating gas dispersal and sensing and computer vision, which modeled the visual representation of a gas plume. It defined the environment, simulated wind and gas dispersion data, and then integrated the results in Unity for plume rendering, sensor simulation, and environment visuals. The improvement could be new functionalities and optimization of the online rendering. Another olfactory navigation based on multisensory integration inspired by the adult silk moth was introduced in [86], which acquired odor and wind direction information and the employees’ virtual reality system. It improved the search success rate compared to the conventional odor search algorithm. Figure 12 provides the odor-source search fields.

Martinez et al. [87] designed an odor recognition system with an SNN; it was based on a bilateral comparison between spatially separated gas sensors. The navigation laws depended on the sensory information from plume structures, which could be performed in a turbulent odor plume. If multiple odor plumes existed, the approach could not locate and identify the sources. Ref. [88] solved the gas source localization problem by two destination evaluation algorithms with gas distribution mapping (GDM), SLAM, and anemotaxis in an unknown GPS-denied environment. The algorithms were anemotaxis-GDM for gas source tracking and a FRONTIER-multicriteria decision-making that determined destination candidates. Even though the success ratio reached 71.1% in the simulation, additional experiments need to be conducted.

## 6. Sound-Based Navigation

Animals also use sound for localization and navigation. Sound-based navigation is usually used for sound-source localization and echo-based navigation. It can perform 3D localization and construction. Sound signal processing is considered during the development. Some AI techniques are applied, such as neural networks, feedback models, and optimized algorithms. The cited papers employ auditory sensors, sonars, ultrasonic sensors, and event-based silicon cochlea.

A bioinspired binaural 3D localization was proposed with a biomimetic sonar, which used two artificial pinnae with broadband spectral cues [89]. It selected the azimuth–elevation pair for 3D localization with a binaural target echo spectrum. Steckel and Peremans [90] developed a sonar-based spatial orientation and construction called BatSLAM, inspired by RatSLAM. It used a biomimetic sonar for navigation in unmodified office environments, which allowed navigation and localization with sonar readings at a given timescale.

Abbasi et al. [91] developed a two-wheeled mobile robot with a trajectory tracking controller and a path recommender system that adopted particle swarm optimization and B-spline curves. It combined ultrasonic sensors and a camera, and the tracking module reduced the sensor error. The system’s limitation was that it could only perform offline. It could not handle real-time tasks, planning, or collision detection. The operation in a cluttered indoor environment was not achieved.

An enhanced vector polar histogram algorithm was proposed for local path planning of a reconfigurable crawling robot with an ultrasonic sensor [11]. It achieved obstacle avoidance with the proposed algorithm but would lead to double back distance due to an erroneous grouping of obstacles. This resulted in the unnecessary movements of the robot. The displacement of ultrasonic sensors was also challenging in rolling mode. Tidoni et al. [92] designed an audiovisual feedback model to improve sensorimotor performance in brain–computer interfaces with human footstep sounds. The audiovisual synchronization decreased the required control time and improved the motor decisions of the user. However, the interactive capabilities with the environment were limited, and the adaptative behavior was not shown.

Ghosh et al. [93] obtained the optimal path with flower pollination and bat algorithms using a fitness function that considered the distance and goal-reaching behavior within unknown environments and by conducting the experiment with an ultrasonic sensor. The flower pollination algorithm depended on different pollination processes of flowering plants, while the bat algorithm relied on frequency tuning and echolocation. The system was only applied to a single robot, and it did not consider the static or dynamic obstacles during operation.

Event-driven neuromorphic sensors include the silicon cochlea and the silicon retina, which encode the sensory stimuli across different pixels as asynchronous streams. A novel preprocessing technique was applied on the output cochlea spikes to better preserve the interspike timing information for recurrent SNN in [94]. Glackin et al. [95] presented an SNN-based model of the medial superior olive. It used the spike-timing-dependent plasticity rule to train the SNN, and it was used for sound localization with an accuracy of 91.82%. The layers included input and cochlea model layers, bushy cell neurons, a graded delay structure and an output layer. However, a limitation was the angular resolution, and the network architecture was restricted to a subset of angles. More frequency ranges and a hardware implementation should be further considered.

## 7. Inertial Navigation

A popular sensor to estimate the robot’s body is the inertial measurement unit (IMU). An IMU is often used for indoor navigation because the indoor environment is GPS-denied. The design of an IMU tends to consist of micro-electromechanical systems (MEMS) sensors with lightweight and small sizes. Filtering techniques are widely used for data fusion with other sensory inputs for reducing errors, such as the extended Kalman filter (EKF). A new mapping and tracking framework was proposed based on parametrized patch models with an IMU and an RGB-D sensor in [96]. It was operated in real time for surface modeling and terrain stepping with a dense volumetric fusion layer and multiple-depth data.

**Figure 13:**
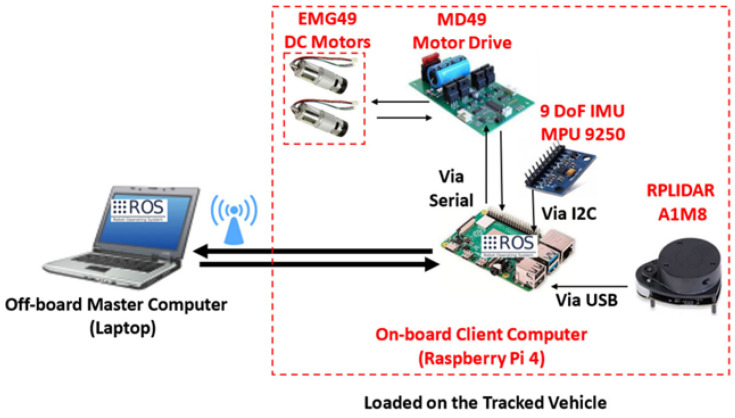
System architecture of the system [97].

Sabiha et al. [97] used teaching–learning-based optimization for online path planning in a cluttered environment, considering the potential collision and path smoothness as a multiobjective optimization problem, as shown in Figure 13. An IMU achieved the robot perception by collaborating with LiDAR and wheel odometry, then via data fusion with the EKF. The model could not adapt to dynamic environments and was limited to convex and regular obstacles. The path planning algorithm should be improved with a vision system to detect the surroundings. Chen et al. [98] used an extended Kalman filter to estimate the spatial motion with a six-axis IMU, joint encoders, and a two-axis inclinometer. The body state also offered sensory information for feedback control, including the damping controller to regulate the body position state in real time.

A reconfigurable rolling–crawling robot was designed for terrain perception and recovery behaviors with an IMU and visual sensor [99], and the remote computer received the video stream in real time to process vision and feedback. However, the autonomous capabilities were limited because the different terrains could not be perceived or classified, and the robot could not walk with stairs. Yi et al. [100] designed a self-reconfigurable pavement sweeping robot with a four-wheel independent steering drive and used a multiobjective optimization and the optimal instantaneous center of rotation. It incorporated multiple sensors, including LiDAR, IMU/GNSS, encoder, and camera, and the optimization considered the distance, varying width, clearance to a collision, and steering. Reconfiguration and modern optimization are needed in the future.

A sensory-perceptual system exploits the environment to identify obstacles, walls, or structures and is regarded as perception-driven obstacle-aided locomotion. Ref. [101] introduced a multipurpose modular snake robot with an IMU, which used a linear discriminant analysis to identify terrain in real time. It remodeled the elastic joint with a damper element and redesigned the joint module using internal hardware components. The environments and gaits were limited in the simulation. The kinematic model and mechanical design should be further developed.

Kim et al. [102] presented a novel millirobot to extend the robot’s perception capabilities with a swarm-sensing module based on a six-axis IMU, a processing and communication module, a locomotion module, and a proximity-sensing module with a camera and proximity and light sensors. Decentralized formation control was used in behavior studies via a swarm-sending module to exhibit collective behaviors, such as dynamic task allocation, chain formation, aggregation, and collective exploration. Higher-performance sensors could be further included in the research. Improvements of the processing power and locomotion capabilities of the robot are also required.

An event-based visual–inertial odometry algorithm was designed to fuse range and event simultaneously to improve the robustness and accuracy of the position in high-dynamic scenes in [13]. It ran in real time with IMU and range measurements processed with a sliding-window optimization by feature extraction and tracking. The shortcoming of the algorithm was that it did not consider the noise and the illumination, which may reduce the accuracy of the position.

## 8. Multimodal Navigation

Multimodal navigation involves several types of approaches to enhance the adaptability and performance of the navigation. This section mainly discusses the combination of sensors and an AI-based approach. Multimodal navigation plays the most important role in the recent development of bioinspired perception and navigation. It focuses on sensor fusion and AI techniques. A popular integration is based on visual sensors. Neural networks have gained the most attention in the research literature. Multimodal navigation is aimed at navigating in challenging environments.

**Figure 14:**
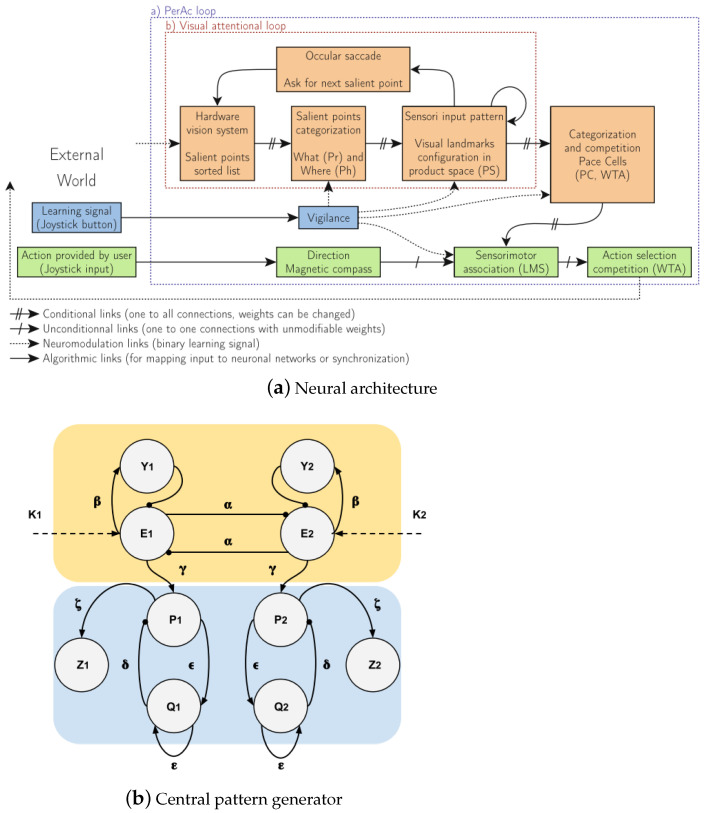
(**a**) Neural architecture in which the loop runs for a visual scene for neural networks with a visual attentional loop to categorize landmark [103]. (**b**) The network of the central pattern generator; arrows refer to the excitatory connections, and lines end at inhibitory connections [3].

An embedded architecture was proposed as a multiscale attentional architecture for bioinspired vision-based indoor robot perception in [103]. Its main layer included the vision attentional layer and the neural control layer inspired by a multimodal approach for fusion and learning, as shown in Figure 14a, which achieved environmental learning while still working on a dynamically configurable camera. From the inspiration of how locusts process visual information, a collision detection algorithm was introduced with a collision-detector neuron even for high-speed vehicles [104]. It reproduced the excitation of the collision-detecting neuron even with a low image resolution and planned the evasive maneuvers. However, the algorithm could not respond well to overhead signs or ground shadows.

An exploration and navigation system was proposed based on animal behaviors as central pattern generators as shown in Figure 14b [3]. The action selection model generated a signal to trigger behaviors for homing, approaching, and exploration, and the path integration model stored the signal for direct movement and the walked path with 90% accuracy. Moreover, the orientation correction model redirected the virtual agent, and the central pattern generator produced the path.However, the most significant shortcoming was that the model lacked any obstacle avoidance capability, which resulted in the model only being able to be implemented in a very simple environment. Porod et al. [105] designed a cellular neural network system with nanoelectronic devices, which mapped the motion detectors and biological features into spatiotemporal dynamics. An improvement should focus on sensory-computing-activating circuits.

A log-polar max-pi model was presented with visual place recognition for a neural representation of the environment by unsupervised one-shot learning in [106]. It processed visual information by two distinct pathways with a novel detector and used the one-shot learning mechanism and the spatial landmark’s position to learn the representation of new positions for high localization scores and performance [106]. The shortcoming of that model was that it recruited new neurons for every landmark, even the learned one, which affected the computation frequency.

Head-direction cells and place cells are gained by visual input and by encoding the orientation and positions of the animal, and the animal’s brain can process raw visual data into high-level information [2]. A spatial cognition model inspired by neurophysiological experiments in rats was proposed, and it integrated head direction cells, hippocampus place cells and entorhinal cortex grid cells to provide spatial localization and orientation [107]. The robot used a world graph topology for navigation in that approach, integrating place cell activation with neural odometry. However, it could not support remapping or long-term navigation. A reuse of the cells was also impossible due to the system’s processing and memory limitations.

A generic neural architecture was conceptualized using an online novelty detection algorithm and visual place cells in [108]. It was able to self-evaluate sensory motor strategies and regulate its behavior for complex tasks, estimating sensory-motor contingencies. Future work mentioned developing a homeostatic mechanism to self-regulate the system. Suzuki and Floreano [109] designed an enactive vision with neural networks for wheeled robot navigation. The network had no hidden units for simplification, and it was evolved with a genetic algorithm and encoded in a binary string. However, the neural architecture was limited and had to be designed for each task carefully.

A spatial association algorithm was proposed for autonomous positioning based on place cells with a vision sensor in [110]. It used a vision sensor to get the distance between the landmarks and the robot to construct the map of place cells, then used a radial basis function neural network to achieve the association and memory of spatial locations. However, it required a limit on the number of landmarks, memory points, and place cells. Yu et al. [111] constructed a rat brain spatial cell firing model. It used an IMU and the robot’s limbs to get the position, then encoded the position information by theta cells and mapped it to place cells with a neural network. The connection weights of the network were adjusted by Hebb learning.

Kyriacou [112] proposed a biologically inspired model based on head direction cells, which implemented an evolutionary algorithm to determine the parameters and then trained the artificial neural network connections. It used vestibular, visual, and kinesthetic inputs incorporated with the objective function. Montiel et al. [113] designed a parallel control model based on an optical sensing system to define the movements and a convolutional neural network (CNN) to analyze the environment and motion strategies to achieve real-time control. It was proposed with two loops as a feedback motion control framework for service robotics related to monitoring and caring for people. Egomotion classifiers were designed with the first CNN for compound eye cameras, and it classified the local motions of each eye image [114]. The voting-based approach was used to aggregate the final classification for 2D directions, and the experiment had an 85% accuracy in the building environment. A limitation of the classifier was that it could recognize backward and forward motions and could only classify 2D movements.

An SNN processes bioinspired information, especially for event-based data. It was proposed to overcome artificial neural networks’ energy limitations, but it also provides synergies with neuromorphic sensors [115]. Event sensors use send-on-delta for temporal sampling scheme to capture environmental information, which is triggered when the signal deviates by delta. Send-on-delta schemes only send new reports when the monitored variable decreases or increases beyond a threshold [116]. Some sensors with an event-driven architecture support send-on-delta monitoring and the send-on-delta concept can reduce reports and save bandwidth. Therefore, it is suitable for wireless sensor networking. Comprehensive surveys of event-based sensors are published in [22,117,118].

An SNN achieves bioinspired bottom-up visual attention, and it restricts the data flow for online processing [119]. The SNN controls the camera’s view to switch to another stimulus and can focus on simple stimuli. The SNN is expected to be evaluated with a higher cognitive phenomenon in the future study. An indoor flying project evolved adaptive spiking neurons with a multistage vision-based microrobot [120]. The adaptive spiking neurons provided matches to the digital controllers, and it explored the space of solutions. However, the model could not be used in unpredictable and changing environments because it could not be adapted on the fly.

Alnajjar et al. [121] offered a hierarchical robot controller based on Aplysia-like SNN with spike-time-dependent plasticity, and each network was stored in a tree-type memory structure. The memory enhanced navigation in previously trained and new environments, and dynamic clustering and forgetting techniques could control the memory size. The sensors used were infrared/light sensors, sound sensors, and cameras. Arena et al. [122] developed a reactive navigation technique with a chaotic system in real time, and a distributed sensory system provided real-time environment modifications. It took inspiration from the olfactory bulb neural activity and used continuous chaos control for the feedback. The experiment was conducted with distance sensors and an FPGA architecture. However, the contextual layer had the shortcoming of dealing with short-term and long-term memory for navigation.

Another FPGA-based framework was proposed for a multimodal embedded sensor system, which incorporated optic flow and image moments in low-level and mid-level vision layers, inspired by mammalians [123]. The computation speed achieved real-time estimation, but the optical flow computation at different moments and hardware implementation were the shortcomings of the method. Elouaret et al. [124] designed a high-performance and low-footprint accelerator for image recognition with a spatial working memory on a multi-FPGA platform. It implemented a bioinspired neural architecture to process visual landmarks and used a distributed version for a multi-FPGA platform. The system could not deliver high performance; a scheduling system or a postscheduling algorithm could be used for improvement.

Sanket et al. [125] proposed a minimalist sensorimotor framework with onboard sensing and monocular camera, including a vision-based minimalist gap-detection model and visual servoing. It was used for finding and go through an unknown gap with a deep learning optical flow, and the parameters would be chosen dynamically in further research. Ref. [126] modeled a looming spatial localization neural network from the inspiration of the Monostratified Lobula Giant type1 neurons with a presynaptic visual neural network. It perceived its looming spatial location and biological counterpart and was sensitive to size and speed. The model interacted with dynamic environments.

Wang et al. [127] designed an optimized dynamical model as a multiscale extension for cognitive map building with grid cells. The robotic application was achieved by combining a vision-assisted map correction mechanism, place cells, and a real-time path integration of grid cells. The system consisted of vision information on depth and RGB data, a multiscale path integration, place encoding, a hierarchical visual template tree, a topological map, and an accumulated error correction. Barrera et al. [128] involved spatial cognition in goal-oriented tasks, and the developed model produced a cognitive map by integrating visual and kinesthetic information. Reinforcement learning and Hebbian learning were used for the training to learn the optimal path leading through the maze. The adaptation could be further improved. The system could not react to the internal changes in the map, such as closing or opening corridors.

Pang et al. [129] trained robots using hybrid supervised deep reinforcement learning (DRL) for the person following with visual sensors, while supporting dynamic environments. Features were extracted from monocular images for a supervised learning (SL) policy network, then the RL policy and value network were applied. However, distance detection should be utilized. Ref. [130] provided synthetic classification methods for terrain classification by a simple-linear-iterative-clustering-based SegNet, which is a deep learning network, and a simple-linear-iterative-clustering-based support vector machine (SLIC-SVM) with visual sensors. The algorithms used the single-input multioutput model to improve the applicability of the classifier and conduct superpixel segmentation on images, while the terrain information could be more focused.

Arena et al. [131] applied a dynamic spatiotemporal pattern for bioinspired control, and sensors provided heterogeneous information in the perceptual core to build the environment. A nonlinear lattice of neuron cells presented the robot’s internal state for many solutions. It implemented the reward-based learning mechanisms and reaction–diffusion cellular nonlinear networks for perception, albeit only for a simple environment. A novel predictive model of visual navigation approaches based on mammalian navigation with a combination of neurons observed in the brain was presented in [16] for visually impaired people. It stored the environment representations as place cells and used the grid-cell modules for absolute odometry and an efficient visual system to perform sequential spatial tracking in redundant environments [16]. It was claimed to be robust by forcing the agent to repeat the learned path, but other cues’ positions would cause interference.

**Figure 15:**
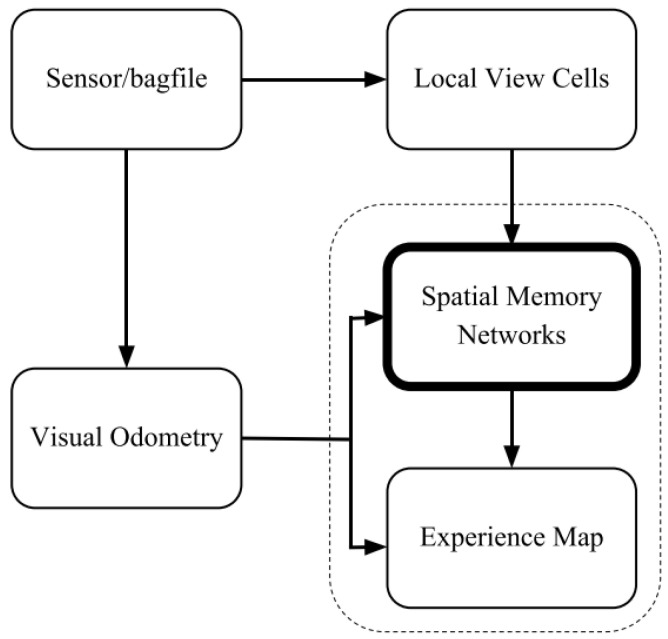
The architecture of the cognitive mapping model and the visual odometry node estimates the velocity, and the spatial memory network performs path integration and decisions [132].

A cognitive mapping model was introduced with continuous attractor networks, conjunctive grid cells, and head direction cells to combine velocity information by encoding movements and space as in Figure 15 [132]. The model was robust for building a topological map with a monocular camera, then using head direction cells and conjunctive grid cells to represent head directions, positions, and velocity, and then using the neural mechanisms for spatial cognition of the brain. The model’s limitations included large numbers of units. They could not provide a metric map [132]. Ref. [133] developed a novel quadrant-based approach based on the grid neuron to input body movement and output periodic hexagonal gridlike patterns. Then, the authors implemented a cognitive map formation with the place–grid neuron interaction system to make predictions. The model provided body parts’ movement tracking for several spatial cognitive tasks, which was better than other grid neuron models. However, it was only implemented in a 2D environment.

Bioinspired place recognition was presented for the RatSLAM system with a modified growing self-organizing map for online learning in unknown environments in [134]. It used a pose cell network for path integration and view cells for the visual association to produce an experience map. It is expected to combine local key-point descriptors with GIST features for hierarchical scene learning in a further study. Another neuroinspired SLAM system was proposed based on multilayered head direction cells and grid cells with a vision system in [135]. The vision system provided self-motion and external visual cues and used a neural network to drive the graphical experience map with local visual cues. Then, it corrected accumulative path integration errors by a multilayered experience map relaxation algorithm.

Ni et al. [136] introduced a bioinspired neural model based on SLAM with an extended Kalman filter (EKF) for searching and exploring. The adaptive EKF used a neural model to adjust the observation and system noise weights to guarantee stability and accuracy. It could also deal with the noise in abnormal conditions.

**Figure 16:**
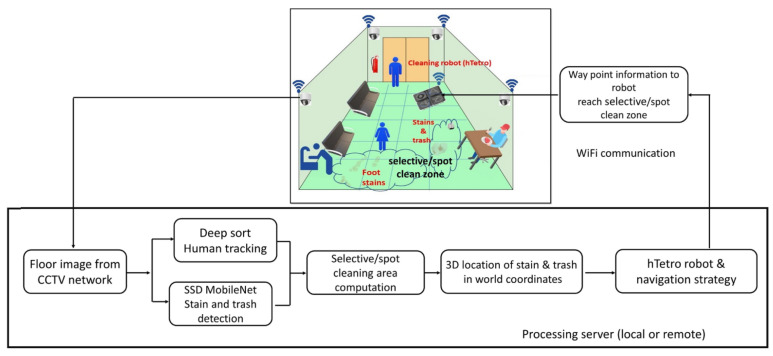
Overview of a selective area-cleaning framework [137].

Ramalingam et al. [137] presented a selective area-cleaning/spot-cleaning framework based on an RGB-D vision sensor and a deep learning algorithm for indoor floor-cleaning robots, as shown in Figure 16. The human traffic region was traced by a simple online and real-time tracking algorithm and the dirty region was detected by a single-shot detector MobileNet. Then, waypoint coverage path planning was achieved via an evolutionary algorithm on the selective area. Ref. [138] developed a deep-network solution for autonomous indoor exploration with several CNN layers in a hierarchical structure. The system used visual RGB-D information as input and provided the main moving directions, and the Gaussian process latent variable mode created the feature map. Online learning algorithms were proposed as the next step of the study, as well as extending the target space to the continuous space.

**Figure 17:**
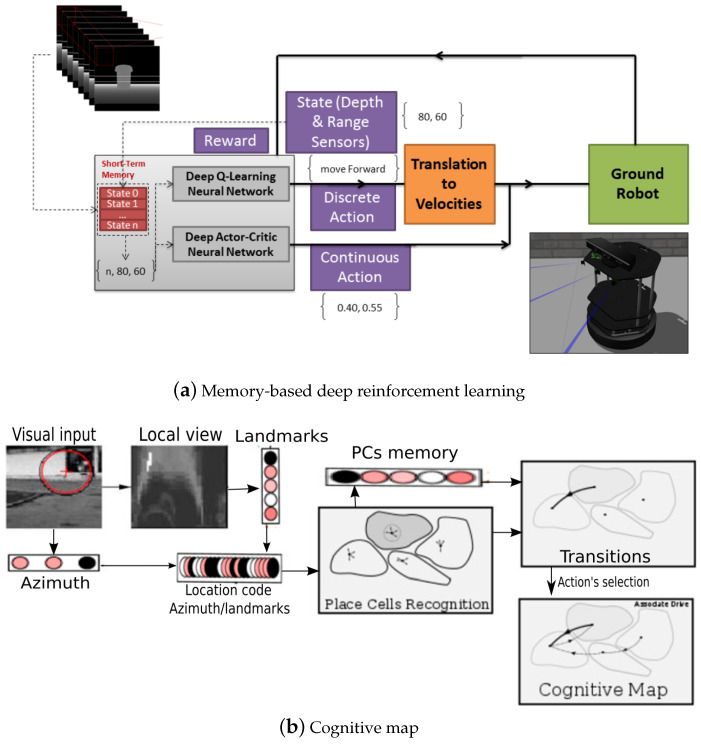
(**a**) The workflow of memory-based deep reinforcement learning [12]. (**b**) The construction of place cells on the cognitive map [139].

A novel learning approach named memory-based deep reinforcement learning was proposed with a centralized sensor fusion technique in [12], which could learn from scratch for exploration and obstacle avoidance without preprocessing sensor data. It considered exploration as a Markov decision process and uses memory-based deep reinforcement learning as in Figure 17a; it had further potential to reduce the search status of robots. Chatty et al. [139] designed a learning-by-imitation method for a multirobot system, building a cognitive map by coupling a low-level imitation strategy. It had a positive effect on the behaviors of human and multirobot systems and on sharing information and individual cognitive map building in an unknown environment. The visual input of place cells was as in Figure 17b.

Another cognitive architecture was described based on a visual attention system in social robots, which used a client/server approach [140]. The attention server sent motion commands to the robot’s actuators, and the attention client gained the common knowledge representation. The cognitive architecture consisted of five hardware tiers, the programming interface, controllers, the operational level, the task manager level, and the high-level mission. The energy consumed could be further analyzed.

The neurocognitive structure is presented in [141], which consists of a hippocampal-like circuitry and a hierarchical vision architecture. The architecture is for spatial navigation, and it combines the hippocampus and a biological vision system as a brain-inspired model, including motor and sensory cortical regions. A more complicated environment and computation complexity would be a further improvement. Kulvicius et al. [142] designed an odor-supported place cells algorithm with a simple feed-forward network, which analyses the olfactory cues on spatial navigation and place cell formation. It uses self-marking for goal navigation by odor patches and a Q-learning algorithm, which supports hierarchical input preference and remapping.

**Figure 18:**
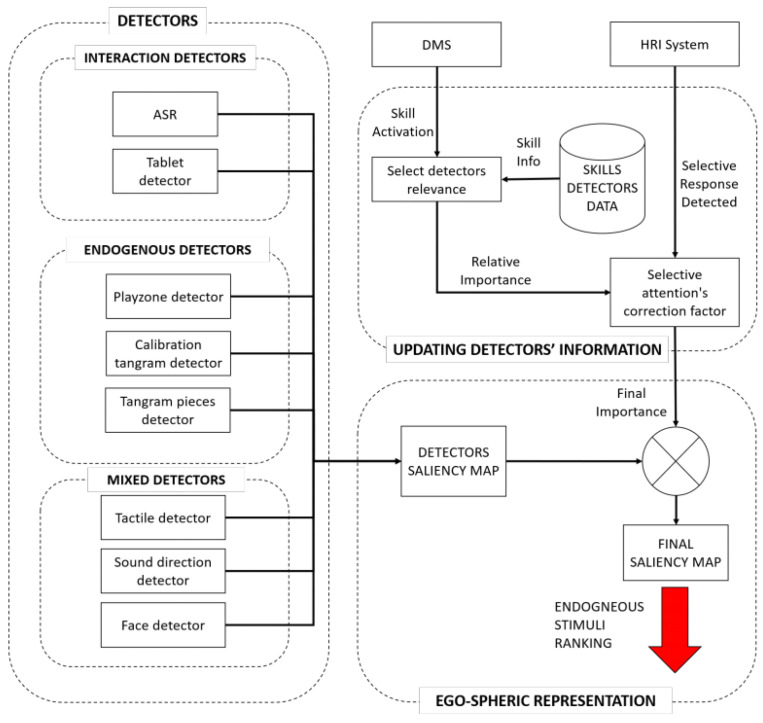
The detectors extract information from the environment that is classified into auditory, visual, and tactile information; the system performs an integration process for multisensory aggregation [143].

An endogenous artificial attention-based architecture was presented with multiple sensory sources, including vision, sound, and touch in [143], as shown in Figure 18. It achieved real-time responses and chose the relevant information for natural human–robot interaction, reacting to sustained and punctual attention, although it required long-term tests in stressful situations. Moreover, the correct functioning of the system should be tested with the target population for performance. Ref. [144] proposed map planning and neural dynamics for unknown dynamic environments, and it utilized an ultrasonic sensor and Dempster–Shafer inference rule, then used a topologically organized neural network. It determined the dynamics of neurons by a shunting equation and considered the uncertainties of sensor measurements.

A multimodal tactile sensing module was proposed for surface characterization with a micro-electromechanical, magnetic, angular rate, and gravity system in [10]. It used a classification method by a multilayer perceptron neural network, but it needed to be evaluated with a larger dataset. Zhang et al. [145] developed a B self-organized fission–fusion control strategy for swarm control, considering dynamic obstacle interference. Their algorithm achieved fission–fusion movement with a control framework and then built a subswarm selection algorithm with a tracking function. However, some specific parameters were not investigated in depth during the experiments.

Yin et al. [146] presented a neurodynamic-based cascade tracking control algorithm for AGV, providing smooth forward velocities with state differential feedback control. The bioinspired neurodynamic model used two levels of controllers with a cascade tracking control strategy for smooth and robust control, although a real experiment would be required. Moreover, lateral and longitudinal slips should be considered for tracking problems. Ref. [147] employed CNNs such as Siamese neural networks in visual-teach-and-repeat navigation for image registration which was robust to changes in environmental appearance. Due to the high efficiency when generating high-fidelity data, real-time performance was achieved. However, the training example was limited during the development.

Dasgupta et al. [148] presented an artificial walking system that combined neural mechanisms and a central pattern generator and had distributed recurrent neural networks at each leg for sensory predictions. It adapted the movement of the legs for searching and elevation control in different environments and used neural mechanisms for locomotion control with real-time data. Ref. [149] described a generic navigation algorithm based on a proximal policy optimization with onboard sensors, which collaborated with long short-term memory neural networks and incremental curriculum learning. The proximal policy optimization reinforcement learning algorithm was optimized for real-time operation, and the recurrent layer supported backtracking when stuck. It could be deployed to search, rescue, or identify gas leaks. However, real-world experiments were not conducted.

Al-Muteb et al. [150] developed autonomous stereovision-based navigation with a fuzzy logic controller in unstructured, dynamic indoor environments. It provided indoor lighting adaptability via point-cloud filtering and stereomatching parameters. It assisted the system with a laser measurement sensor for path adjustment and emergency braking to move through waypoints. An intelligent system was proposed for robot navigation with an adaptive-network-based fuzzy inference system which added the fuzzy logic to the neural network and then used the ant colony method in a continuous environment as the second layer [151]. The employed robots had two infrared sensors and a displacement device, then used five layers of the fuzzy system: adaptive, rule, normalization, and defuzzification layers, and a single node.

A dynamic recurrent neurofuzzy system was improved with a short memory and ultrasonic sensors to avoid obstacles by supervised learning in [152]. The second layer of the system was the feedback connection to memorize the previous environment data, and the structures and parameters were automatically optimized. Nadour et al. [153] introduced a hybrid type-2 fuzzy logic controller with optical flow based on an image processing and video acquisition algorithm. The optical flow used the Horn–Schunk algorithm to estimate the velocity, and the fuzzy logic controller consisted of a fuzzification, a rule-based process, an inference mechanism, a defuzzification, and a type reducer.

Ref. [154] employed a multilayer feed-forward neural network with infrared and ultrasonic sensors as an intelligent controller in a dynamic environment. It inputted obstacle distance to the target angle and position, respectively, and produced the steering angle. The cognitive tasks were handled by the four-layer neural network with the time and path optimization.Ref. [155] presented a winnerless competition paradigm, and the spatial input determined the sequence of saddle points of the path and then reflected the spatial–temporal motion. The framework was an action-oriented perception based on Lotka–Volterra system with cellular nonlinear networks and distance sensors. The challenges of the work included real and complex environments, bioinspired learning methods for SLAM, and a neural-model-based EKF.

A hybrid rhythmic–reflex control method was developed based on oscillatory networks and feedback information, in which real-time joint control signals provided adaptive walking for a biped robot [156]. The walking pattern was realized in real time with the body attitude angle. The limitation of the work was that it only applied to the sagittal plane, although the antidisturbance ability and irregular terrains are important for the robot. Pathmakumar et al. [157] designed an optimal footprint-based coverage system for false-ceiling inspection with a multiobjective optimization algorithm. The system included a localization module with UWB, a controller module with Wi-Fi, locomotion modules with encoders and motors, and a perception module with the camera. The robot followed a zigzag path planning strategy to maximize the coverage area. However, dynamic optimization and energy consideration were not determined, and static and dynamic obstacles were not considered.

## 9. Others

Corrales-Paredes et al. [158] proposed an environment signaling system with radiofrequency identification (RFID) for social robot localization and navigation. It used the human–robot interaction to get information for the waymarking or signaling process, and it successfully experimented in a structured indoor environment. The robot could not learn from the environment or possible changes. Other environments were considered possible improvements, such as museums, hospitals, shopping centers, etc.

Collective gradient perception was enhanced based on the desired speed and distance modulations for a robot swarm in [159]. It used social interactions among robots to follow the gradient with an ultrawideband (UWB) sensor, assisted by a laser distance sensor, and a flow camera. It could be used for searching and localizing sources, but it would need more sensors to sense light, temperature, or gas particles.

Le et al. [160] introduced a multirobot formation with a sensor fusion of UWB, IMU, and wheel encoders within a cluttered unknown environment. The global path planning algorithm incorporated skid-steering kinematic path tracking, and the dynamic linearization decoupled the dynamics to control the leader robot of the formation. However, the experiment was only conducted in static environments.

## 10. Discussion and Conclusions

From the literature, vision-based, remote sensing, tactile-sensor-based, olfactory, sound-based, inertial, and multimodal navigation are the main bioinspired perception and navigation systems for service robots in indoor environments. They are inspired by animals such as rats, bats, and mammalian, etc. Environmental information is gained through different sensors, depending on the applications or surroundings. The vision-based approaches are the most popular among these methods, as well as in combination with other approaches for multimodal navigation.

More precisely, Table 1 lists the vision-based approaches with their contributions, sensors, and real-time performance, and the most popular method is the optic flow, which is used by bees. The vision-based applications include transport [9,18,39,46,50,60], exploration [8,19,24,26,40,41,45,49], tracking and assist [31,56,58], security and surveillance [7,52], homing and searching [5,54,55], floor cleaning [6,43], and search and rescue [44]. Only 43.75% of the cited papers indicate they can operate in real time, which is achieved by processing or computation speed, minimal computation load, or predefined parameters. The challenges of visual navigation include not having an optimal path or dynamic obstacles, more parameters or coefficients that should be considered, the accuracy in the navigation, an optimal implementation, limited assumptions, and more sensors or approaches required.

Additionally, Table 2 displays papers about remote sensing navigation, focusing on LiDAR, laser ranger, and infrared sensors. The tasks include exploration [63,64,66,71], wheelchair [78], transport [65,73,74,75], fire services or search and rescue [70], floor cleaning [68], tracking [76], education [72], and caring and service [79]. Some approaches are combined with path planning algorithms, including metaheuristic algorithm, potential field, and A* for navigation [66,68,70,78]. Online performance only reaches 29.41% in remote sensing perception with models, algorithms, or controllers. The weaknesses of the cited remote sensing techniques include unfiltered points, safety issues, real implementation, multirobot exploration, sensors’ limitations, control parameters, sensing systems, and environmental adaptability.

Table 3 compares tactile-sensor-based, olfaction sensor-based, sound-based, and inertial navigation. Tactile sensor-based approaches with online performance utilize board processing, feedback, or technologies. Future work on tactile-based approaches is about improving accuracy, and the applications are autonomous transport and perception [14,81,82,83]. Olfaction-based navigation approaches are implemented for transport [15,84,86,87], gas dispersion [85], and gas source localization [88]. The limitations of olfactory navigation include obstacle avoidance techniques, active searching, several odor plumes, and precise localization. The sound-based approaches focus on sonar or ultrasonic sensors. The primary tasks are exploration [89,92] and transport [11,90,91,93]. Some approaches are combined with metaheuristic algorithms for efficiency, such as [91,93]. The challenges are sensor displacement, sensor fusion, online collision detection and planning, and a dynamic environment.

Inertial navigation usually is applied for reconfigurable robots [99,100,101,102], and some approaches uses sensor fusion techniques [96,97,98]. Inertial navigation can be utilized for exploration [96,99], sweeping [100], and autonomous transport [97,101]. Other navigation implements RFID or UWB for localization for entertainment [158] and searching [159]. The drawbacks of the research include optimization reconfiguration, autonomous capabilities, performance, sensor fusion, dynamic environment with dynamic obstacles, and the kinematic model. The online performance of the sound-based and inertial navigation systems is achieved via an algorithm or the model efficiency. Improving the framework for sensors and operating within dynamic environments are considered future work.

Moreover, Table 4 lists the multimodal navigation. The applications of the multimodal navigation consist of entertainment [143], security [133], transport [2,106,107,110,112,147], assistance [2,16,108], exploration [3,134,135,138,141], social applications [12,140], tracking [105,125,131,145,146], caring and monitoring [113], disaster monitoring or search and rescue [136,149], floor cleaning [137], wheeled robot [109], person following [129], and false-ceiling inspection [157]. The combination of virtual sensors and neural networks is most commonly used in multimodal navigation, which represents 77.19% of the cited research.

Furthermore, 59.65% of the navigation approaches can perform real-time feedback due to the AI-based approach, such as learning algorithm, neural network, fuzzy logic, and optimization. Hardware, such as the architecture and controller, achieves some of the online performance. Challenges exist in multimodal navigation including complex environments, remapping and reusing different environments, reducing computational resources, undesired responses, learning datasets, cognitive phenomenon, parameter settings, layer or neuron design, energy, accuracy, dynamic obstacles, and real experiments.

From the bioinspired perception and navigation review, the main applications include autonomous transport, exploration, floor sweep, and search and rescue. These strategies allow service robots to operate safely, estimating their states and positions relative to their surroundings. Multimodal navigation offers real-time performance due to the AI-based approach, and it combines with other sensors for perception. The most popular collaboration is with visual sensors and neural networks.

In the real implementation of robot perception and navigation, indoor environments are dynamic and changing, including moving objects or people, challenging lighting [25], and stairs or carpets [99]. The dynamic obstacles are unpredictable and hard to avoid. However, most papers do not consider dynamic environments, except [13,136,154], which tried to solve the problem with learning approaches. Some studies indicate a dynamic environment as future work [13,93,97,124,157]. However, the problem of moving objects or dynamic obstacles is still not solved. Navigation in dynamic environments requires real-time performance, high adaptability, a quick decision-making ability, and object detection and avoidance.

Learning ability and adaptability are also future research directions. The trend of bioinspired perception is moving towards multimodal approaches, which are expected to provide real-time responses [132,143]. The learning ability enables a robot to use previous information to train the model to process new information and quickly respond to changes in surroundings. Neural networks and machine learning are taken into account for learning strategies, such as SNN [119], reinforcement learning [12], CNN [105], attention mechanism [143], etc. Continual detection and avoidance algorithms should also be considered. Fault detection and tolerance frameworks are expected to be developed in future research.

Sensor fusion is one of the main directions of research, which incorporates several types of sensors, such as combining visual, tactile, and auditory sensors [143], a tactile model with nine-DOF MEMS MARG [10], IMU and visual sensors [96,102], more multimodal approaches (refer to Section 8), etc. Because a single sensor easily gains some bias, other sensory inputs can be used to correct these errors. A great sensor fusion algorithm can provide accurate localization and navigation to determine the robot’s orientation and position. The dynamic and unpredictable environment requires high accuracy to locate the robot and its surroundings. It is also crucial for swarm operation.

Future research also focuses on swarm intelligence, which consists of multiple robots in large groups. Swarm navigation allows robots to execute complicated tasks, explore unknown areas, and improve efficiency. The communication between swarm individuals and the kinematics of different robots are significant challenges [61]. The sensor-based communication protocols must be addressed in physical swarm systems [62]. The issues of the optimization of the navigation algorithm, decision-making strategy, energy consumption, and safety are essential for deployment [66,73]. The swarm size, behavior, and coordinated movements must also be considered [75].

Real-world experiments remain a challenge [50,75,109]. Future research should test and validate approaches in different complex environments, not just be restricted to a specific or simple environment. The representation of the cells and obstacle should consider irregular shapes [97,132,156]. Hardware limitations and computational performance also limit the deployment of bioinspired models [72,123]. It is challenging to integrate the developed approaches into a suitable robot with the required sensors successfully.

## Figures and Tables

**Table 1:** Vision-based navigation.

Paper	Contribution	Sensors	Real Time	How to Achieve Real-Time Operation
[9]	Resource-efficient vision-based navigation	Optic flow	Real time	Optimized parallel processing on FPGA
[18]	Extract the depth structure from the optic flow	Optic flow	N/A	
[19]	A quantitative model of the optic flow	Optic flow	N/A	
[31]	A robust gradient-based optical flow model	Optic flow	Real time	Lightweight operating system
[29]	Time-of-travel methods	Optic flow	N/A	
[20]	Lobula plate tangential cells	Optic flow	N/A	
[7]	A gradient-based optical flow model	Optic flow	Real time	GPU speed
[24]	A novel integrated, single-chip solution	Optic flow	Real time	Computation speed
[23]	Hierarchical SNN	Optic flow, event-based camera	Real time	Large-scale SNNs
[25]	Self-supervised optical flow	Optic flow, event-based camera	Real time	Self-supervised neural network
[26]	Control systems based on insects’ visuomotor control systems	Optic flow	N/A	
[38]	Highly efficient computer vision algorithm	Optic flow	N/A	
[161]	An actor–critic learning algorithm	Optic flow	N/A	
[17]	A miniature hovercraft	Optic flow	N/A	
[30]	Feedback loops	Optic flow	N/A	
[28]	Attitude can be extracted from the optic flow	Optic flow	N/A	
[33]	Ultralight autonomous microfliers	Optic flow, gyroscopes, anemometer, Bluetooth	N/A	
[34]	Visuomotor control system	Optic flow	N/A	
[35]	Minimalistic motion vision	Optic flow	N/A	
[162]	Learning process	Optic flow	N/A	
[37]	Optic flow-based autopilot	Optic flow	N/A	
[27]	Adaptive peripheral visual system	Optic flow	N/A	
[36]	Wide-field integration of optic flow	Optic flow	N/A	
[8]	A Portable bioinspired architecture	Visual	Real time	A minimal quantity of resources
[48]	A self-adaptive landmark-based aggregation method	Landmark	N/A	An error threshold parameter
[51]	Landmark-tree (LT) map	Landmark and omnidirectional camera	N/A	
[49]	A landmark vector algorithm	Landmark-based	N/A	
[5]	Entropy-based vision and visual topological maps	Landmark and entropy-based vision	Real time	A conventional bug algorithm/metric maps
[50]	Pan–tilt-based visual sensing system	Landmark and visual sensor	Real time	Perception-motion dynamics loop
[39]	A bioinspired SLAM algorithm	SLAM and monocular or stereovision systems	Real time	CPU-GPU architecture
[47]	Hierarchical look-ahead trajectory model (HiLAM)	SLAM	Real time	RatSLAM
[40]	State estimation pipeline	SLAM	Real time	Standard frames
[41]	Refocused events fusion	SLAM	Real time	Throughput
[42]	Artificial neural SLAM framework	SLAM, event-based camera	N/A	
[45]	Movement-based autonomous calibration techniques	SLAM, cameras, sonar sensors, a laser, an RGB, and a range sensor	Real time	Online sensor fusion
[43]	Dirt-sample-gathering strategy/ACO	SLAM	Real time	Dirt-gathering efficiency
[44]	An decentralized approach	SLAM	N/A	
[46]	Environmental-adaptability-improved RatSLAM	SLAM	N/A	
[6]	Visual navigation	Depth camera	Real time	Superpixel image segment algorithm
[57]	Generate robot actions	Depth camera	Real time	Partially observable Markov decision process
[32]	Ocular visual system	Ocular sensor and Monte Carlo	N/A	
[53]	Distributed wireless nodes	Wireless vision sensors and mosaic eyes	Real time	Image acquisition and processing module
[54]	Parallel mechanism-based docking system	Camera infrared sensor	Real time	Stereo camera
[55]	Signal processing and control architectures	Visual tool	Real time	Custom OpenGL application
[56]	Mobile robots cooperation	Camera	Real time	Visual servoing
[52]	Intelligent recognition system	Wireless camera	Real time	Path delineation method
[58]	Visual-Teach-and Repeat	Visual servo	N/A	
[59]	Reconfigurable biomimetic robot	Monocular vision	Real time	GPGPU coding
[60]	Efficient stereoscopic video matching	Stereoscopic vision	N/A	
[61]	Modeling multirotor UAVs swarm deployment	Visual	N/A	
[62]	V-shaped formation control	Binocular	N/A	

**Table 2:** Remote sensing navigation.

Paper	Contribution	Sensors	Real Time	How to Achieve Real-Time Operation
[63]	Wide-field integration (WFI) framework	LiDAR-based	N/A	
[64]	Modular robot	LiDAR-based	Real time	Robot framework
[78]	Tree expansion using particle swarm optimization	LiDAR-based	Real time	World model update
[65]	LiDAR-Based Local path planning	LiDAR-based	N/A	
[66]	Metaheuristic salp swarm algorithm and deterministic coordinated multirobot exploration	LiDAR-based	Real time	Metaheuristic algorithms
[70]	Social potential field framework	LiDAR-based	N/A	
[68]	Modified A-star algorithm	LiDAR-based	Real time	Modified A-star algorithm
[71]	Motion policy; a direct mapping from observation to control	Laser ranger and omnidirectional	N/A	
[67]	Evolutive localization filter (ELF)	Laser ranger	N/A	
[69]	Chemical signaling	Infrared sensors	N/A	
[73]	Generic fault-detection system	Infrared proximity sensors	N/A	
[75]	Self-organized aggregation behavior	Infrared sensors	N/A	
[76]	Cognitive dynamic system	Radar and sonar	N/A	
[77]	Steering a swarm of robots	Proximity, infrared, ultrasonic sensors, and light-dependent resistor	N/A	
[74]	A behavioral algorithm	Ultrasonic and infrared sensors and light-dependent resistor	N/A	
[72]	Robotic platform	Short-range infrared proximity sensors	N/A	
[79]	Control mechanisms	Distance sensor	Real time	ESP32 microcontroller

**Table 3:** Tactile sensor-based, olfaction sensor-based, sound-based, and inertial navigation approaches.

Paper	Contribution	Sensors	Real Time	How to Achieve Real-Time Operation
[14]	A hybrid obstacle-overcoming method	Tactile sensor-based	Real time	Signal is transferred on board
[81]	Triboelectric nanogenerators	Tactile-sensor-based	Real time	Palm structure and triboelectric nanogenerator technology
[82]	Feedback control	Tactile-sensor-based and whisker	Real time	Whisker feedback
[83]	Terrain-recognition-based navigation	Tactile-sensor-based and whisker	Real time	On-board reservoir computing system
[80]	Motor learning	Tactile-sensor-based	Real time	Nonlinear recurrent SNN
[15]	Odor-tracking algorithms with genetic programming	Olfaction-sensor-based	N/A	
[84]	Search based on the silkworm moth	Olfaction-sensor-based	N/A	
[86]	Multisensory-motor integration	Olfaction-sensor-based and visual	N/A	
[87]	Odor recognition system	Olfaction-sensor-based and an SNN	N/A	
[85]	Gas dispersal and sensing alongside vision	Olfaction-sensor-based	N/A	
[88]	FRONTIER-multicriteria decision-making and anemotaxis-GDM	Olfaction-sensor-based, gas distribution mapping (GDM), and anemotaxis/SLAM	N/A	
[89]	Binaural sonar sensor	Sound-based/sonar	N/A	
[92]	Audiovisual synchrony	Sound-based/Visual	N/A	
[11]	Enhanced vector polar histogram algorithm	Sound-based, ultrasonic sensors	N/A	
[91]	A two-wheeled mobile robot with B-spline curves and PSO	Sound-based, ultrasonic sensors/camera	N/A	
[90]	Sonar-based spatial orientation	Sound-based, sonar	N/A	
[93]	FPA and BA metaheuristic	Sound-based, ultrasonic sensors	Real time	Algorithm efficiency
[95]	SNN-based model	Sound-based	N/A	
[96]	Curved patch mapping and tracking	IMU and RGB-D	Real time	Parametrized patch models
[99]	Reconfigurable rolling–crawling robot	IMU and visual sensor	Real time	Remote computer for vision processing and feedback
[100]	4WISD reconfigurable robot	IMU, Velodyne, and LiDARs, ultrasonic sensors, absolute encoder, wire encoder, and camera.	N/A	
[102]	Millirobot with an open-source design	IMU and camera	N/A	
[97]	Teaching–learning-based optimization and EKF	IMU, wheel odometry, and light detection and ranging (LiDAR)	Real time	Algorithm
[101]	Multipurpose modular snake robot	IMU	Real time	Linear discriminant analysis
[98]	Sensor data fusion algorithm	IMU, a 2-axis inclinometer, and joint encoders	Real time	Overall control strategy
[158]	Environment signaling system	Radiofrequency identification (RFID)	N/A	
[159]	Collective gradient perception	UWB, laser, and camera	N/A	
[160]	Multirobot formation with sensor fusion-based localization	UWB position system, IMU, and wheel encoders	Real time	Sensor fusion

**Table 4:** Multimodal navigation.

Paper	Contribution	Sensors	Real Time	How to Achieve Real-Time Operation
[143]	A Bioinspired endogenous attention-based architecture	Virtual	Real time	Selective attention’s correction
[110]	Spatial association	Virtual, neural network, place cells	Real time	Neural network
[133]	Quadrant-based approach	Virtual, neural network, place cells	N/A	
[2]	Slow feature analysis	Vision-based, place cells and head direction cells	N/A	
[107]	Spatial cognition model	Vision-based, place cells, grid cells and head direction cells	N/A	
[16]	Navigation inspired by mammalian navigation	Vision-based	Real time	Prediction-oriented estimations
[132]	Cognitive mapping model	Virtual, neural network, head direction cells, conjunctive grid cells, SLAM	Real time	Conjunctive space-by-movement attractor network
[112]	Biologically inspired model, evolutionary algorithm	Virtual, neural network, head direction cells	N/A	
[106]	Log-polar max-pi (LPMP)	Virtual, neural network	Real time	Visuospatial pattern
[103]	Embedded control system	Vision-based, Neural control layer	Real time	Neural control layer
[104]	Collision detection	Virtual, Neural network	Real time	Collision-detector neuron in locusts
[3]	Central pattern generators (CPGs)	Virtual, neural network	Real time	Pattern generators
[10]	Tactile probe	Tactile Sensing	N/A	
[134]	Place recognition, RatSLAM system	Virtual, Neural network	Real time	Self-organizing neural network
[12]	Deep-reinforcement-learning-based intelligent agent	Virtual, neural network, infrared proximity sensor	Real time	Memory-based deep reinforcement learning
[13]	Range and event-based visual-inertial odometry	Virtual, inertial odometry, range	Real time	Sensor
[135]	NeuroSLAM	Virtual, neural network	Real time	Neural network
[108]	Generic neural architecture	Virtual, neural network	Real time	Online detection algorithm
[144]	Neural dynamics and map planning	Virtual, neural network	Real time	Neural network
[139]	Learning by imitation leads	Virtual, cognitive map	Real time	Cognitive map
[145]	Self-organized fission–fusion control	Multimodal	N/A	
[146]	Neurodynamics-based cascade tracking control	Multimodal	N/A	
[105]	Nanosensor-Enhanced CNN	Virtual, neural network	N/A	
[131]	Dynamic spatiotemporal patterns	Virtual, neural network	N/A	
[119]	Bioinspired visual attention process using SNNs	Virtual, neural network	Real time	Restrict the data flow
[114]	CNN-based egomotion classification framework	Virtual, neural network, compound eye	N/A	
[125]	Minimalist sensorimotor framework	Virtual, deep learning	Real time	Minimalist philosophy
[141]	Vision-enhanced neurocognitive structure	Virtual, neural network	N/A	
[147]	Contrastive learning	Virtual, neural network	Real time	High efficiency
[122]	Multisensory integration	Virtual, distance sensor	Real time	FPGA architecture
[123]	FPGA-based embedded sensor system	Virtual, optic flow	Real time	Processing speed
[124]	Bioinspired neural architecture	Virtual, image	Real time	FPGAs
[113]	Parallel control model	Virtual, image	Real time	Two loops form
[148]	Distributed recurrent neural network	Leg, neural network	Real time	Adaptive locomotion
[150]	Stereovision-based navigation system	Virtual, fuzzy logic	Real time	Algorithm
[153]	Type-2 Fuzzy logic	Virtual, fuzzy logic	N/A	
[149]	Generic navigation algorithm	Neural network, onboard sensor	Real time	Proximal policy optimization
[151]	Intelligent system, ACO	Infrared sensor, fuzzy logic	N/A	
[154]	Multilayer feed-forward neural network	Infrared sensor, ultrasonic, neural network	Real time	Neural controller
[140]	Visual attention system	Virtual, cognitive architecture	N/A	
[137]	Selective area-cleaning/spot-cleaning technique	Virtual, deep learning	Real time	SSD MobileNet
[127]	Optimized dynamical model	Virtual, grid cells	Real time	Vision-assisted map correction mechanism
[126]	Looming spatial localization neural network	Virtual, motion-sensitive neuron	N/A	
[138]	A novel deep learning library	Virtual, RGB-D information, deep learning	Real time	Deep learning
[120]	Vision-based microrobot	Virtual, adaptive spiking neurons	N/A	
[109]	Enactive vision	Virtual, neural networks, computer vision	N/A	
[155]	Winnerless competition paradigm	Neural networks, olfactory	N/A	
[136]	A bioinspired-neural-model-based extended Kalman filter	Neural networks, SLAM	Real time	Neural dynamic model
[142]	Odor-supported place cell model	RL, olfactory	N/A	
[156]	Hybrid rhythmic–reflex control method	Neural network	Real time	ZMP-based feedback loop
[128]	Spatial memory and learning	Visual, cognitive map	N/A	
[152]	Dynamic recurrent neurofuzzy approach	Ultrasonic, learning	Real time	Fuzzy logic
[130]	Simple-linear-iterative-clustering-based support vector machine (SLIC-SVM), simple-linear-iterative-clustering-based SegNet	Visual, SVM	Real time	Sensor
[129]	Hybrid supervised deep reinforcement learning	Visual, RL, Markov decision process (MDP)	Real time	SL policy network training
[157]	Optimal functional footprint approach	Visual, camera, beacon, UWB, encoder, motor, Wi-Fi	N/A	
[121]	A hierarchical autonomous robot controller	Visual, infrared, sound, neural network	N/A	
[111]	Brain spatial cell firing model	IMU, neural network	N/A	

## Data Availability

Not applicable.

## Abbreviations

The following abbreviations are used in this manuscript:

SLAM	Simultaneous localization and mapping system
ACO	Ant colony optimization
HSI	Hue, saturation, and intensity
HiLAM	Hierarchical look-ahead trajectory model
APF	Artificial potential field
PF	Particle filter
PSO	Particle swarm optimization
GDM	Gas distribution mapping
IMU	Inertial measurement unit
EKF	Extended Kalman filter
SLIC-SVM	Simple-linear-iterative-clustering-based support vector machine
SL	Supervised learning
DRL	Deep reinforcement learning
RFID	Radiofrequency identification
UWB	Ultrawideband
MDP	Markov decision process
AER	Address-event representation
SNN	Spiking neural network
CNN	Convolutional neural network
MEMS	Micro-electromechanical systems
